# Feature Paper in Environmental Chemistry and Technology

**DOI:** 10.3390/ijerph182010874

**Published:** 2021-10-16

**Authors:** Daniela Varrica

**Affiliations:** Dipartimento di Scienze della Terra e del Mare (DiSTeM), Via Archirafi 22, 90123 Palermo, Italy; daniela.varrica@unipa.it

Attention to the environment and its problems has undergone unprecedented growth in recent years. The credit is mainly due to a reinterpretation of the relationship between humans and the environment, which has favored a change in attitudes towards environmental problems and has opened new opportunities for scientific–technological reflection.

This awareness has prompted many researchers and governments of the most industrialized countries to confront the extraordinary development of technological innovation and environmental issues. Human beings live in the realm of nature and interact with it constantly. The influence of nature on human life manifests itself in different forms, including the air we breathe, the water we drink, the food we eat, etc. Therefore, any change in the environment can not only cause devastating effects for nature but can also pose a threat to humans.

The theme of the human–environment relationship can be traced back to the presence of humans on Earth, but the degree of environmental destruction that communities are witnessing can be considered very new. From the very beginning, humans have modified the surrounding environment to make it more conducive to their own survival, as evidenced by a study carried out at the Col du Dome glacier that highlighted a phenomenon of heavy-metal pollution in western Europe associated with emissions from mining and smelting operations during European antiquity (about 5000–6000 years BP) [1].

It is now known that the current level of impact of human activities on the environment cannot be maintained without causing irreversible damage to life on Earth. There has been a long debate on the relationship between economic growth and the environment, especially in the second half of the twentieth century. Several studies have highlighted an exchange between the improvement of the lifestyle of modern society and the consequent negative impact on the environment. A first study on the link between economic growth and environmental damage was expressed in the book *Limits to Growth* [2]. In this book, the authors asked themselves questions: What do we want our world to be like? Can we continue to continuously expand production and consumption? The questions are still relevant.

The modern debate on sustainable development has focused more on the potential complementarity between growth and the environment, where growth must be considered compatible with better environmental quality [3]. Contamination from metals, metalloids, and organic components has increased significantly around the world due to the increase in urbanization and industrialization. Their negative environmental impacts are evident in terms of air, water, and soil pollution; therefore, it is now necessary to try to combine the environment around us with technological progress. As a great writer wrote, “*The world is a beautiful place and worth fighting for*” (Ernest Hemingway). Therefore, environmental chemistry and technology must be widely used to develop new analytical techniques to determine the presence of pollutants in different environmental spheres (water, soil, and air), providing important support to environmental protection agencies. The Special Issue *Feature Paper in Environmental Chemistry and Technology* was intended to provide the scientific community with innovative approaches to environmental protection, with the aim of evaluating (a) the behavior and transport of certain chemicals in the various environmental spheres (atmosphere, hydrosphere, and lithosphere) and (b) the possibility of developing new technologies for environmental protection.

From the four articles published in the Special Issue, it is evident that the study of the environment is not the heritage of a single discipline but requires the contribution of knowledge ranging from geology to chemistry and from biology to engineering.

Canova et al. [4] performed determinations of As, Cd, Co, Cr, Cu, Fe, Hg, Mn, Ni, Pb, Se, and Zn on the feathers of three species of Ardehydes, to evaluate a possible direct effect of these elements on the survival and growth rate of the chicks. The authors concluded that the conservation status of heron populations in northern Italy is probably more affected by other factors, such as climate changes, an altered aquatic environment, and, consequently, food quality.

The interaction between humans and nature is certainly to be found in the mists of time of the human era, developed by humans for their own survival. Among the main processes of interaction, agricultural activity is certainly one that has influenced and modified the morphology of the land. A study conducted in China, in Guizhou’s Yinjiang County [5], showed that the REE content in secondary forest land and shrub profiles was mainly regulated by the soil pH and Fe content, while the clay content and agricultural activities were the main controlling factors in the soil profile of abandoned farmland. This study highlights the role of agricultural activities in influencing the distribution of REEs in karst soils, which could provide some insights into protecting the soil environment.

Technological advancement led humans to mix minerals with polyvinyl chloride (PVC). I refer to the wide use of vinyl asbestos floors that have been used for almost half a century, for flooring public buildings, schools, hospitals, and housing, due to their mechanical characteristics, low cost, rapid installation, and easy cleaning. Asbestos added to the aggregates and PVC improved the mechanical, heat, and corrosion resistance properties. Since 1990, the use of asbestos has been banned in Italy, as it is considered a carcinogenic mineral, but the Italian Ministerial Decree (MD) 06/09/94 indicates asbestos vinyl tiles as non-friable materials and, therefore, as posing few risks to human health. A study carried out by Zichella et al. [6] aimed to determine if asbestos floor tiles, after decades of use, maintained their characteristics of compactness and non-friability by using three different experimental tests.

Increasingly, we hear of new emerging micropollutants, which are defined as “*chemicals that do not have regulatory status*” and “*can have a negative impact on the environmental and human health*”. A particular reference is environmental contamination from pharmaceutical products, which is now becoming an emerging problem. The scientific literature suggests that drugs are widespread contaminants, entering the environment from a myriad of scattered points. The main reservoirs are certainly superficial waters and groundwaters, as well as soils. Therefore, in recent years, several studies involving the use of some metal–organics to remove these new forms of pollutants from the environment have been conducted. In a study conducted by Capsoni et al. [7], two different zinc-based metal–organic frameworks (MOFs) were investigated to remove one of the most used fluoroquinolone antibiotics, ofloxacin (OFL), from polluted water. The obtained removal efficiencies, of 88% for ZIF-8 and 72% for Zn_3_ (BTC)_2_, make these materials promising candidates for removing fluoroquinolone antibiotics (FQs) from polluted waters, notwithstanding their limited reusability in tap water.

The conclusion drawn from the topics addressed in this Special Issue is certainly a reflection of the main environmental problems that can be addressed through technological development. Therefore, the rapid environmental changes that we observe in this historical moment must support a more intense interaction between environmental chemistry and technology to provide important means to environmental protection bodies. Moreover, humans must be an integral part of the environment and not the main modification factor of the ecosystem.

## Data Availability

Not applicable.
